# Rediscovery and systematics of the enigmatic genus *Helicostoa* reveals a new species of sessile freshwater snail with remarkable sexual dimorphism

**DOI:** 10.1098/rspb.2023.1557

**Published:** 2024-01-10

**Authors:** Le-Jia Zhang, Zi-Ang Shi, Zhe-Yu Chen, Thomas von Rintelen, Wei Zhang, Zheng-Jie Lou

**Affiliations:** ^1^ Museum für Naturkunde, Leibniz-Institut für Evolutions- und Biodiversitätsforschung, Berlin, 10115, Germany; ^2^ State Key Laboratory of Protein and Plant Gene Research, School of Life Sciences, Peking University, Beijing 100871, People's Republic of China; ^3^ Department of Life Sciences, Imperial College London, London SW7 2AZ, UK; ^4^ Department of Life Sciences, Natural History Museum, London SW7 5BD, UK; ^5^ Peking-Tsinghua Center for Life Sciences, Academy for Advanced Interdisciplinary Studies, Peking University, Beijing 100871, People's Republic of China; ^6^ Hangzhou Changzheng High School, Hangzhou 310011, People's Republic of China

**Keywords:** sessility, bithyniidae, molecular phylogeny, freshwater ecology, evolutionary innovation

## Abstract

*Helicostoa sinensis* E. Lamy, 1926 is a unique freshwater gastropod species with a sessile habit. This enigmatic species was first found cemented on river limestones from China about 120 years ago and described together with the genus. It was never collected again and has been considered monotypic. Here, we report the rediscovery of *Helicostoa* from several rivers in China, and describe a second species of this genus based on a comprehensive study. In addition to the unique sessile habit of both species, the new *Helicostoa* species presents one of the most remarkable cases of sexual dimorphism within molluscs. Only the adult female is sessile and the original aperture of the female is sealed by shell matter or rock, while an opening on the body whorl takes the function of the original aperture. The male is vagile, with a normal aperture. Our results confirm the recently suggested placement of *Helicostoa* within the family Bithyniidae. The sessility of *Helicostoa* species is considered as an adaption to the limestone habitat in large rivers. The extreme sexual dimorphism and secondary aperture of females are considered as adaptations to overcome the obstacles for mating and feeding that come with a sessile life style.

## 1. Introduction

Sexual dimorphism is widespread and common in animals. Sexual selection, fecundity selection and ecological causation are the three popular explanations for the evolutionary mechanism of sexual dimorphism [1]. Within molluscs, sexual dimorphism is mostly reflected in size differences (e.g. in land snails [2], freshwater snails [3] and marine bivalves [4]); only in a few cases is sexual dimorphism found in shell structure (e.g. in marine [5] and freshwater snails [6]); in some rarer cases, such as the cephalopod *Argonauta* or the Puelche oyster [7], sexual dimorphism extends to striking difference in life history.

*Helicostoa sinensis* is a species of freshwater gastropod described based on about 260 snails cemented to two pieces of limestone collected from China in around 1900. The syntopic sessile mussel *Limnoperna fortunei* (Dunker, 1857) confirmed that they were collected from a freshwater environment [8]. It was assigned to a new family of ‘ taenioglossate prosobranchs' gastropods, Helicostoidae, based on a study of opercula, dry soft tissue and radulae left in the shell [9]. The shells cemented to these limestones displayed two completely different morphotypes: the large, flat ‘P type’ and the small, spiral ‘T type’. The ‘P type’ is much more numerous on the syntype limestones than the ‘T type’ (ratio of at least 4 : 1). Pruvot-Fol suggested that these two morphotypes probably represent different sexes of the same species, viz. a case of sexual dimorphism, despite the lack of any anatomical evidence [9]. Bequaert and Clench considered that the radula and soft parts of this species were similar to that of the species of Valvatidae or Bulimidae (today Bithyniidae) [10]. This species was more recently assigned to the sessile marine snail family Vermetidae Rafinesque, 1815 [11] or kept in Helicostoidae within superfamily Truncatelloidea [12].

Helicostoidae was considered as a monotypic family of Mollusca endemic to China. Neither were the two types of *H. sinensis* collected again since their discovery more than 120 years ago, nor was any additional species of this family found. All specimens of this species were collected from a city called ‘Kouei-Tchéou’ near Yangtze River, more than 1200 km away from Shanghai [8], probably referring to the Three Gorges region [13]. However, no specimen of *H. sinensis* was found during comprehensive surveys of freshwater molluscs in the Three Gorges region of Yangtze River in 1980s [14] and in 1990s [15], which were conducted prior to the construction of the Three Gorges Dam.

Based on a study of historical DNA of the ‘P type’ syntype, Wilke *et al.* proposed that *H. sinensis* should be a subfamily-level taxon within the family Bithyniidae, and Helicostoidae is a synonym of Bithyniidae [16], thus finally clarifying the phylogenetic position of this species. *H. sinensis* is conservatively classified as ‘Critically Endangered’ due to the habitat loss in Three Gorges region and no collecting record for more than 120 years.

In March of 2022, we accidentally rediscovered the living ‘T type’ of this enigmatic sessile freshwater snail in a large tributary of the Pearl River Basin in Guangxi, China, which is far from the Yangtze River (at least 550 km to the main stream). Follow-up surveys led to the discovery of more populations of this species from other tributaries of Pearl River Basin. We conducted a comprehensive study on the morphology, anatomy, phylogeny, and ecology of this species and compared the fresh samples with historic museum collections. Based on molecular phylogeny and shell morphology, we found that ‘T type’ and ‘P type’ should be different species of *Helicostoa*, instead of representing different sex morphotypes of one species *H. sinensis* as proposed by Pruvot-Fol [9]. However, we indeed found a remarkable sexual dimorphism in ‘T type’. Here, we revise the taxonomy of *Helicostoa* and describe ‘T type’ as a new species *Helicostoa liuae* sp. nov. based on an integrative taxonomic approach. The evolutionary innovations, sessility, sexual dimorphism and distribution of *Helicostoa* are discussed in this paper.

## 2. Material and methods

### (a) Materials

**Figure 1.**
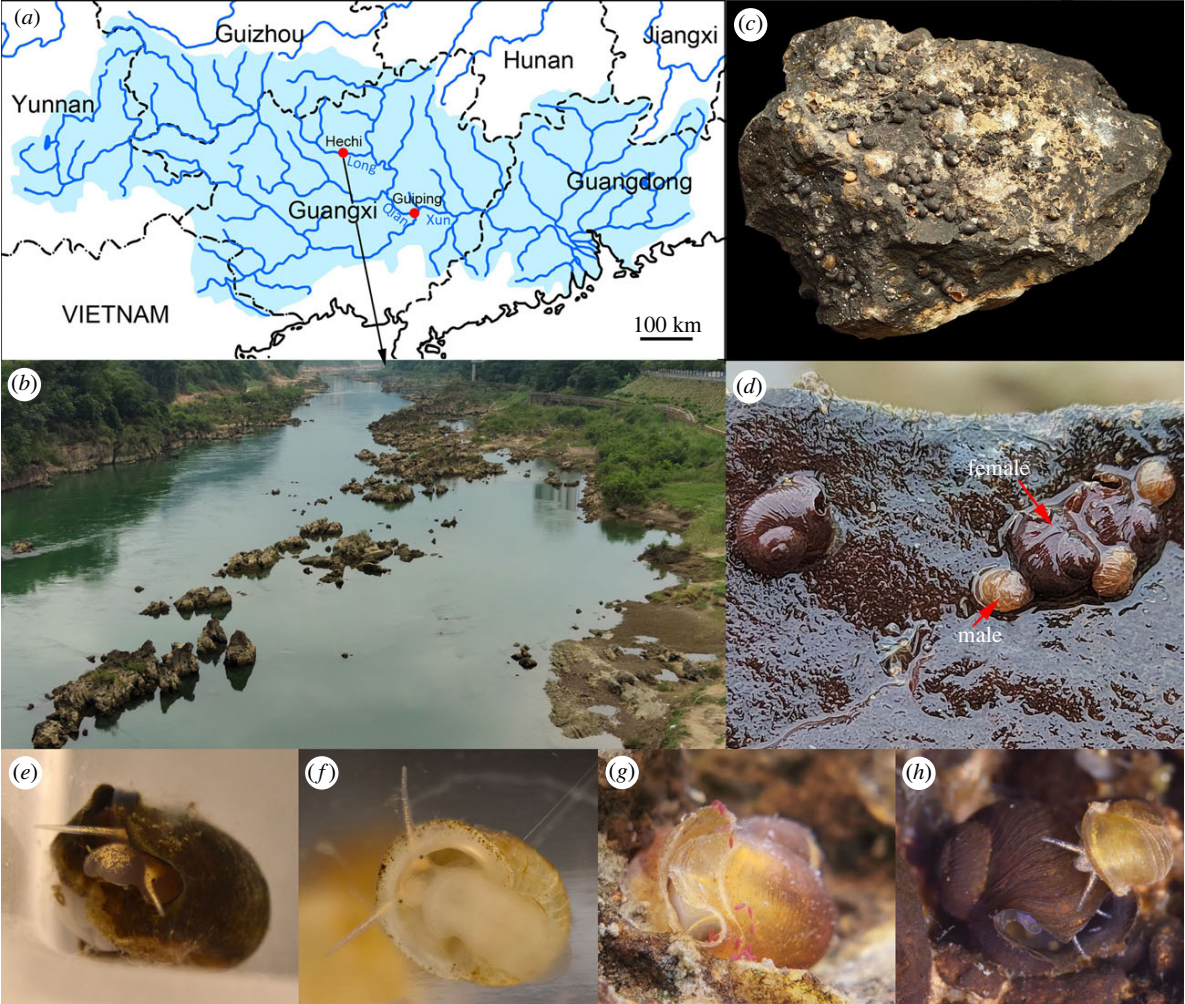
Specimen collecting sites in the Pearl River Basin of China, habitat and living animals in the aquarium for *Helicostoa liuae* sp. nov. (*a*) Map of the Pearl River Basin (light blue area) and the localities of two collection sites (red dot), Hechi (type locality) and Guiping in Guangxi, China. (*b*) Environment of the type locality in Long River, Yizhou, Hechi City, with many large limestones in the river. (*c*) The limestone where the holotype was collected. (*d*) The living animals of females and males *in situ* on the limestone just taken from the river. (*e*) A female detached from stone. (*f*) A vagile male. (*g*) A sessile female in excretion after feeding on red *Chlamydomonas reinhardtii* algae, photographed by Min Chen. (*h*) A male climbing on a female, displaying the remarkable difference in size.

The specimens were collected in three field trips in Long River, Hechi, Guangxi, China during May, July and October 2022, and bought from a local fisherman near the junction of Qian and Xun River, Guiping, Guangxi, China in September 2022 (figure 1). The snail-carrying limestones were collected by hand from a depth of 0.5 metres in the Long River. The sessile specimens used for this study were peeled off from the limestones and kept in 70% ethanol. Some living animals were collected and kept in aquaria in Beijing and Shanghai for several weeks to months. The holotype (female) and the allotype (one designated male paratype) are kept in the collection of Zoological Museum of China, Institute of Zoology, Chinese Academy of Sciences, Beijing (IZCAS), other paratypes are kept in the collection of Museum of Biology Peking University, Beijing (PKU), Museum für Naturkunde, Berlin (ZMB), Muséum national d'Histoire naturelle, Paris (MNHN) and private collection of Zhe-Yu Chen (CZY) and Zi-ang Shi (SZA).

### (b) Examination of morphology

The specimens were photographed in consistent orientation using focus stacking methods with a Sony alpha 7R 4 camera or Canon 5D Mark IV camera attached with a Laowa 25 mm f/2.8 2.5-5X Ultra Macro lens, and a Nikon SMZ18 or Leica M205C stereomicroscope. NIS-Elements D-software and LAS X-software were used to do measurements and focus stacking. The following dimensions were measured on the photos to a precision of 0.1 mm: height (H) and width (W) of shell, height (AH) and width (AW) of aperture, width of opening on body whorl (WO). The WO was measured at the opening (see electronic supplementary material, figure S2*b*). Number of whorls (N) was counted to a precision of ¼ whorls. The protoconch was photographed in detail with Sony alpha 7R 4 camera and the Mitutoyo M-Plan APO 10× Lens. Radulae were extracted through dissection, cleaned by boiling in 1% NaOH solution for half an hour, and rinsed with distilled water. Shell of the attached part, radulae and operculum were coated with gold before scanning electron microscopy (SEM) with FEI Helios Nanolab G3 UC, FEI Quanta 450 FEG or Zeiss *EVO LS10* scanning electronic microscope. The snail bodies, male genitals and egg capsules were taken out during dissection and photographed under a stereomicroscope (Leica MDG34 or Nikon SMZ18). The operculum has been described according to Zhang & von Rintelen [17].

### (c) DNA extraction and amplification

DNA was extracted from the animals of altogether seven individuals from Hechi and Guiping, using a CTAB/chloroform extraction or alkaline lysis protocol. Two mitochondrial genes, cytochrome c oxidase subunit I (COI) and 16S rDNA (16S), were targeted, and partial sequences were amplified through polymerase chain reaction (PCR) with the use of the following primer pairs: LCO1490, 5′-GGTCAACAAATCATAAAGATATTGG-3′ [18] and COX-B7R, 5′-ACCACCAGCTGGATCAAAAA-3′ [19] for COI; 16Sar-L, 5′-CGCCTGTTTATCAAAAACAT-3′ and 16Sbr-H, 5′-CCGGTCTGAACTCAGATCACGT-3′ [20], for 16S . PCR amplifications were conducted in volumes of 25 µL under the following cycling conditions: initial denaturing step at 95°C for 2 min (for COI) or for 3 min (for 16S), followed by 35 cycles of 95°C for 45 s (for COI) or 30 s (for 16S), 57°C for 45 s (for COI) or 52°C for 30 s (for 16S), and 72°C for 30 s, with a final extension step of 10 min at 72°C. Sequencing was conducted by Macrogen Europe, Amsterdam, Netherlands and Tsingke Biotechnology, Beijing, China.

### (d) Phylogenetic analysis

Altogether 14 sequences (7 for each gene) of *H. liuae* have been uploaded into Genbank. The dataset was complemented by two sequences of *H. sinensis* from the historic specimens from Wilke *et al.* [16] and 26 additional sequences that were selected based on the phylogeny from Wilke *et al.* [16] and Bunchom *et al.* [21] (electronic supplementary material, table S1). Sequences were aligned using the Muscle algorithm [22] as implemented in Geneious Prime 2020 (https://www.geneious.com). The genetic distances were calculated using MEGA X [23]. The data set was tested in MEGA X for the best-fit model of sequence evolution by means of the Akaike and Bayesian information criteria. GTR + G + I (for COI) and GTR + G (for 16S) were suggested as the best-fitting nucleotide substitution models, which were employed in the phylogenetic analyses using Maximum likelihood (ML) and Bayesian inference (BI). A ML analysis was conducted using RAxML [24] as implemented in Geneious Prime2020 with 1000 bootstrap replicates. The BI analysis was conducted using MrBayes 3.2.6 [25] as implemented in Geneious Prime 2020 with four independent chains for 5 000 000 generations, samplefreq = 1000, and burnin = 25%.

## 3. Results

### (a) Genetic differentiation and phylogeny

The p-distance of COI between *H. liuae* and *H. sinensis* is approximately 2.3% to 2.4%; the p-distance of COI within *H. liuae* is approximately 0 to 0.8%. The p-distance of 16S between *H. liuae* and *H. sinensis* is approximately 2.1%; the p-distance of 16S within *H. liuae* is approximately 0 to 0.4%.

**Figure 2.**
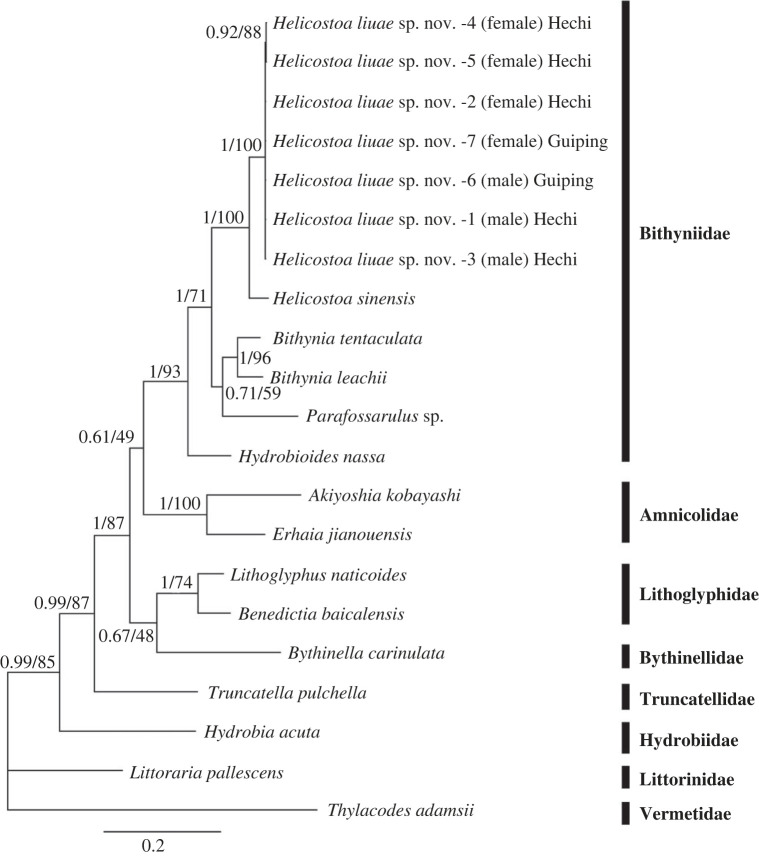
The BI tree showing the phylogenetic position of genus *Helicostoa* within Bithyniidae based on concatenated sequences of cytochrome c oxidase subunit I (COI) and 16S rDNA. Numbers above branches are BI posterior probabilities/ML bootstrap values.

The phylogenetic trees reconstructed by BI and ML are highly congruent in topology, therefore only the BI tree is shown (figure 2). The molecular phylogeny places *Helicostoa* within the family Bithyniidae with high support (figure 2). All sequenced individuals of *H. liuae*, irrespective of sex or geography, form a highly supported clade. *Helicostoa* is monophyletic and *H. liuae* is the sister species of *H. sinensis*.

### (b) Scanning electron microscopy of the attached part

The attached part of *H. liuae*, which should be the original aperture of shell, is sealed by shell matter or rock. Scanning electron micrograph images of the attached part revealed that many foreign objects were embedded into the shell during the development and cementation of the snail (electronic supplementary material, figure S1). On the surface of the attached part of the shell of specimen 1, many long micro-tubes were found clustering together (electronic supplementary material, figure S1*a*–*i*). The micro-tubes seemed soft and smooth. The diameters of micro-tubes were all about 5 µm and their length reached 50 to 60 µm. The cross section of the micro-tube (electronic supplementary material, figure S1*e*) and area with micro-tubes (electronic supplementary material, figure S1*f*–*g*) showed that these tubes were embedded into the shell, which displayed a different microstructure in comparison to areas of the shell without foreign objects (electronic supplementary material figure S1*h*). On the surface of the attached part shell of specimen 2, no micro-tube like those on specimen 1 were present, but many irregular particles (electronic supplementary material, figure S1*j*–*l*). Similar irregular particles were also observed from the attached parts of two additional specimens.

### (c) Systematics

**Superfamily Truncatelloidea Gray, 1840**

**Family Bithyniidae Gray, 1857**

***Helicostoa* E. Lamy,** **1926**

**Type species:** *Helicostoa sinensis* E. Lamy, 1926, by original designation.

**Diagnosis:** shell 2.68 to 11.80 mm in width, broad protoconch, 2.5 to 4 whorls, teleoconch whorls with dense fine ribs, for the sessile individuals on the middle of the last half whorl upper end and lower end of outer lip extending and merging together, enclosing an opening, seam between both ends of outer lip clearly visible.

**Taxonomic remarks:** *Helicostoa sinensis* used to be considered as the only species of the genus *Helicostoa*, which was assigned to the monotypic family Helicostoidae Pruvot-Fol, 1937. However, both Wilke *et al.* [16] and our molecular phylogeny support a position of *Helicostoa* within Bithyniidae, and Helicostoidae must consequently be regarded as a junior synonym of Bithyniidae. Our study of operculum, radula and genital characters supports this taxonomic revision as well. There are two known species of *Helicostoa*. The description of *Helicostoa sinensis* is a redescription incorporating our new results.

***Helicostoa sinensis* E. Lamy, 1926**

**Lectotype:** MNHN-IM-2000-33309, ‘P type’, ‘Koué-Tchéou, ville située sur le Yang Tsé Kiang, à plus de 1200 kms de Chang-Hai’.

**Paralectotypes:** MNHN-IM-2000-38619, 204 specimens of ‘P type’, on the same limestone of the lectotype; MNHN-IM-2000-38620, 60 specimens of ‘P type’.

**Figure 3.**
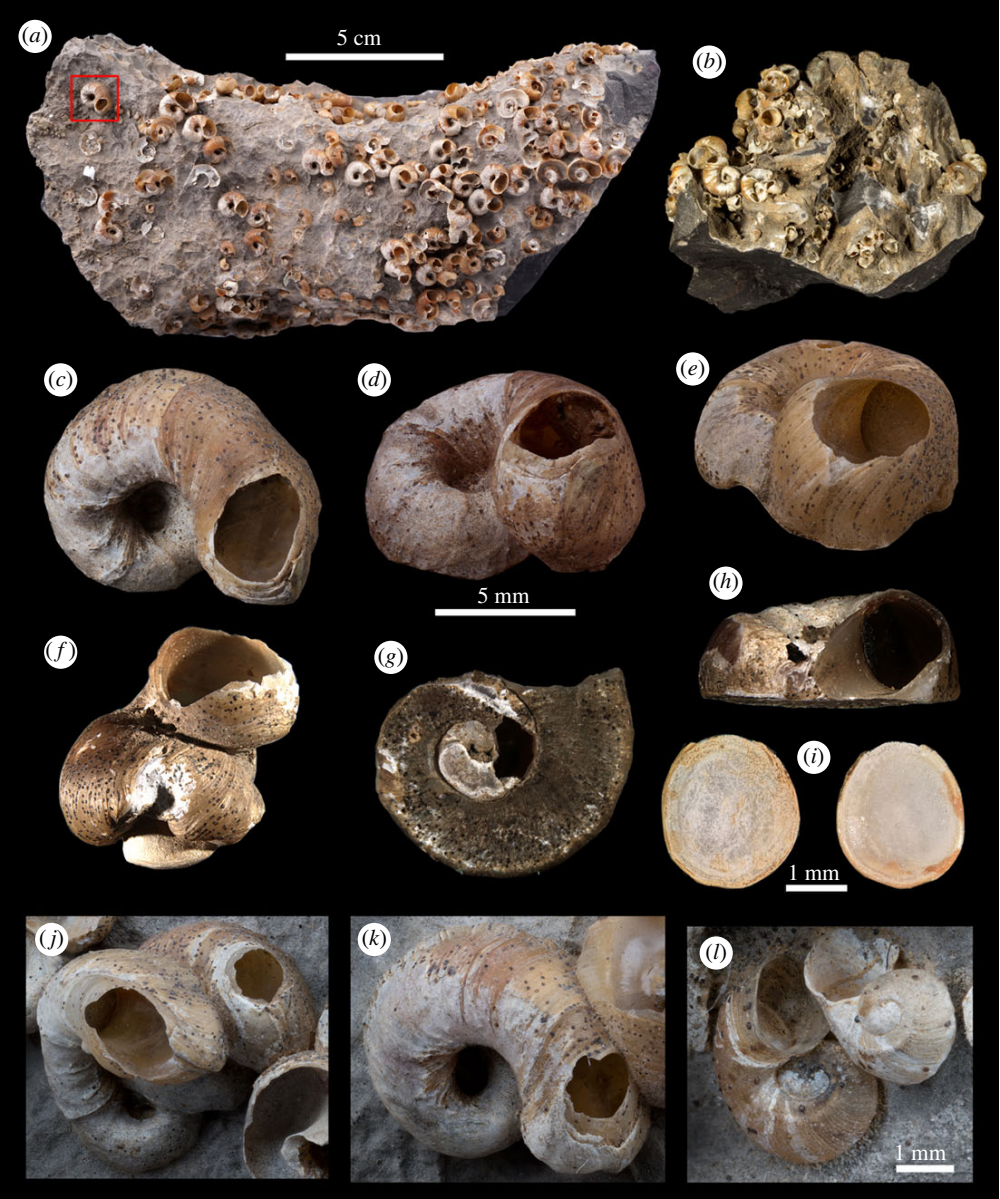
Types of *Helicostoa sinensis* E. Lamy, 1926. (*a*) The stone bearing specimens; lectotype MNHN-IM-2000-33309 is in the red frame. (*b*) The stone bearing specimens; MNHN-IM-2000-38620 (credit: MNHN-Manuel Caballer 2017). (*c*–*d*) Details of MNHN-IM-2000-33309, lectotype. (*e*) A paralectotypes showing the details of seam under the opening. (*f*) An open-coiled paralectotype. (*g*) The base of a peeled paralectotype showing the sessile part. (*h*) The side view of a paralectotype showing the flat base. (*i*) Operculum, left is exterior surface and right is interior surface. (*j*–*k*) paralectotypes *in situ* on rock. (*l*) Juveniles showing the protoconch. (*a*) Scale bar 5 cm; (*c–h*) scale bars 5 mm; (*i*) scale bar 1 mm; (*l*) scale bar 1 mm.

**Description:** shell (figure 3, table 1) 9.30 to 11.80 mm in width, discoidal, orange yellow to light red in colour; usually with 3 inflated whorls, protoconch broad smooth, teleoconch whorls with dense fine ribs, with one obvious keel on the upper part of second whorl; body whorl turning upward, covering spiral whorls partly, and turning downward to attach the substrate, sometimes open-coiled due to obstacle, the edge of body whorl attaching the rock sometimes extended, forming a narrow flat ridge, the area attaching the substrate flat; on the middle of the last half whorl upper end and lower end of outer lip extending and merging together, enclosing a large round or oval opening, seam between both ends of outer lip clearly visible; original aperture completely sealed by shell and attached rock.

**Table 1.** Shell measurements of *Helicostoa* species. Values are arithmetic mean (mm) followed by standard deviation except for number of whorls. Males of *H. liuae* do not have the opening. Aperture of *H. sinensis* is not visible and openings of *H. sinensis* are mostly broken. Therefore, WO of *H. liuae*, AH, AW and WO of *H. sinensis* are not recorded.

species	gender	H	W	AH	AW	WO	W/H
*H. liuae* (*n* = 24)	female	4.71 ± 0.41	5.08 ± 0.52	2.61 ± 0.48	3.45 ± 0.40	0.66 ± 0.10	1.08 ± 0.08
*H. liuae* (*n* = 10)	male	1.96 ± 0.65	2.70 ± 0.84	1.49 ± 0.51	1.71 ± 0.58	/	1.38 ± 0.08
*H. sinensis* (*n* = 10)	unknown	5.14 ± 0.64	10.52 ± 0.89	/	/	/	2.06 ± 0.20

Operculum (figure 3*i*) calcareous, nearly round, thin, transparent white in colour; on exterior surface the nucleus not clearly visible, with many scales on central part and concentric growth line on outer region; on interior surface a large inner opercular region visible.

**Morphological comparisons:** see in *Helicostoa liuae* sp. nov.

**Ecology:** this species was only found on the surface of limestones in rivers.

**Distribution:** this species was only known from the type locality ‘Koué-Tchéou, ville située sur le Yang Tsé Kiang, à plus de 1200 kms de Chang-Hai’, viz. ‘Kouei-Tchéou, city located on the Yangtze River, more than 1200 km away from Shanghai.’ ‘Kouei-Tchéou’ in French may refer to ‘夔州’ (Kuizhou, current Fengjie County near Three Gorges region) or ‘贵州’ (Guizhou Province).

***Helicostoa liuae* Zhang, Shi & Chen sp. nov.**

**LSID urn:lsid:zoobank.org:act:07CE74EC-AEC9-4A54-8883-DCED59B513A6**

**Holotype:** adult female (IZCAS-FG-609823) collected on 8 May 2022 by Xu-Cheng Wei from limestone in Long River, under Long River Bridge, Yizhou District, Hechi City, Guangxi Zhuang Autonomous Region, China.

**Paratypes:** 1 adult male designated as allotype (IZCAS-FG-609824), 16 adult females (5 IZCAS, 5 PKU, 3 SZA, 3 CZY), 17 adult males (7 IZCAS, 8 PKU, 1 SZA, 1 CZY), 1 limestone with 9 adult females and 7 adult males (IZCAS), 1 sub-adult male (CZY), 8 sub-adult females (3 IZCAS, 5 CZY), all collected on July 18, 2022 by Zi-Ang Shi from limestone in Long River, Yizhou District, Hechi City, Guangxi Zhuang Autonomous Region, China (24.5020206°N, 108.6306600°E); 3 adult females (ZMB), 4 adult females (CZY), all collected on May 8, 2022 by Xu-Cheng Wei from the limestone in Long River, Yizhou District, Hechi City, Guangxi Zhuang Autonomous Region, China; 6 adult females (4 IZCAS, 2 PKU), 3 adult male (1 IZCAS, 2 CZY), 1 adult female (CZY), all collected by local people in the junction of Qian, Yu and Xun River in Guiping City, Guangxi Zhuang Autonomous Region, China; 1 adult female (MNHN-IM-2000-38621, ‘T type’), 15 adult females (MNHN-IM-2000-38622, ‘T type’).

**Etymology:** the species is named in honour of Yue-Ying Liu, the late pioneer researcher of Chinese freshwater molluscs.

**Figure 4.**
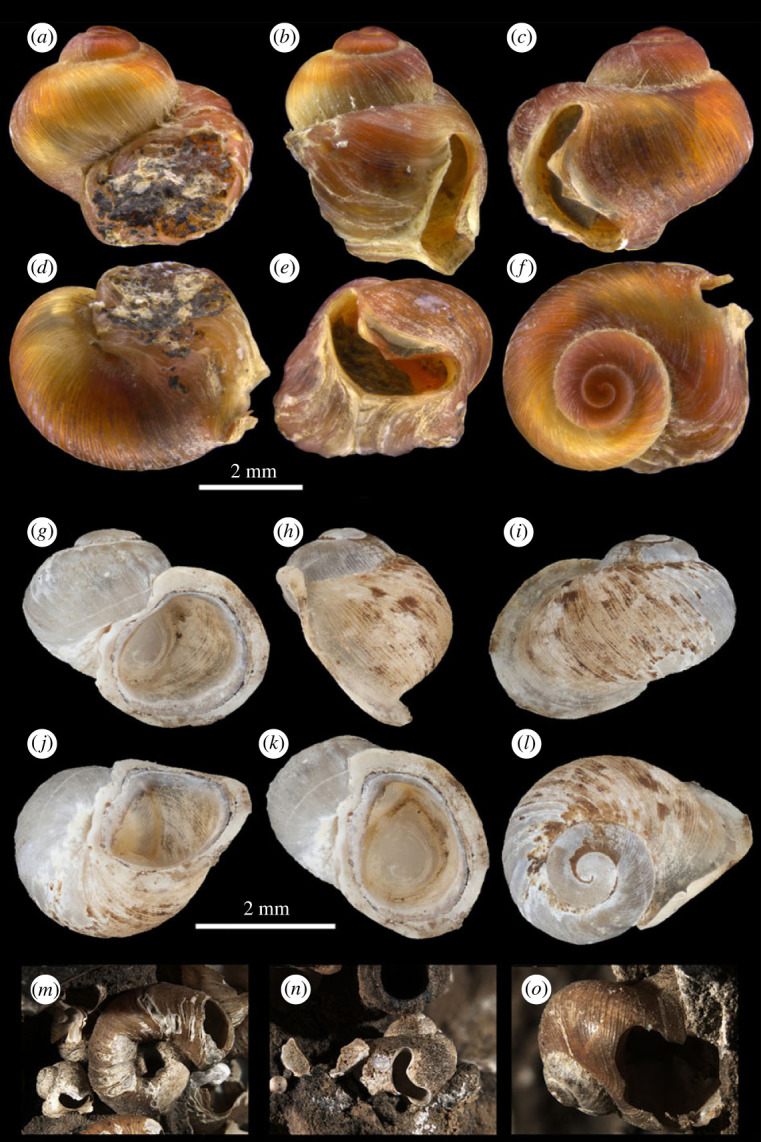
Shell of *Helicostoa liuae* sp. nov. (*a*–*f*) Holotype, IZCAS-FG-609823, adult female, with same scale bar 2 mm. (*g*–*l*) Allotype, IZCAS-FG-609824, adult male, with same scale bar 2 mm. (*m*–*o*) Paratypes *in situ*, MNHN-IM-2000-38622, adult females, (*m*) showing the coexisting of two *Helicostoa* species on one rock.

**Description:** shell of adult female (figure 4*a*–*f*; electronic supplementary material, figure S2*a*–*f*; table 1) 4.17 to 6.75 mm in width, globose, thin but solid, orange yellow to red in colour; low spiral, most with around 3.5 to 4 inflated whorls, protoconch broad and smooth, teleoconch whorls with dense fine ribs; on the middle of the last half whorl upper end and lower end of outer lip extending and merging together, enclosing a wide bow-shaped opening, convex edge of the bow-shaped opening close to the original aperture, seam between both ends of outer lip clearly visible; original aperture large, ovate, but sealed by shell and attached rock, foreign objects sometimes embedded into sealed aperture, inner lip extending and covering umbilicus.

Shell of adult male (figure 4*g*–*l*; electronic supplementary material, figure S2*g*–*n*; table 1) 2.61 to 3.51 mm in width, oblate, thin but solid, pale yellow to orange yellow in colour; low spiral, most with 2.25 to 3 inflated whorls, protoconch broad and smooth, teleoconch whorls with dense fine ribs; aperture large, ovate, labral margin obviously extended, umbilicus mostly covered by inner lip, sometimes open.

**Figure 5.**
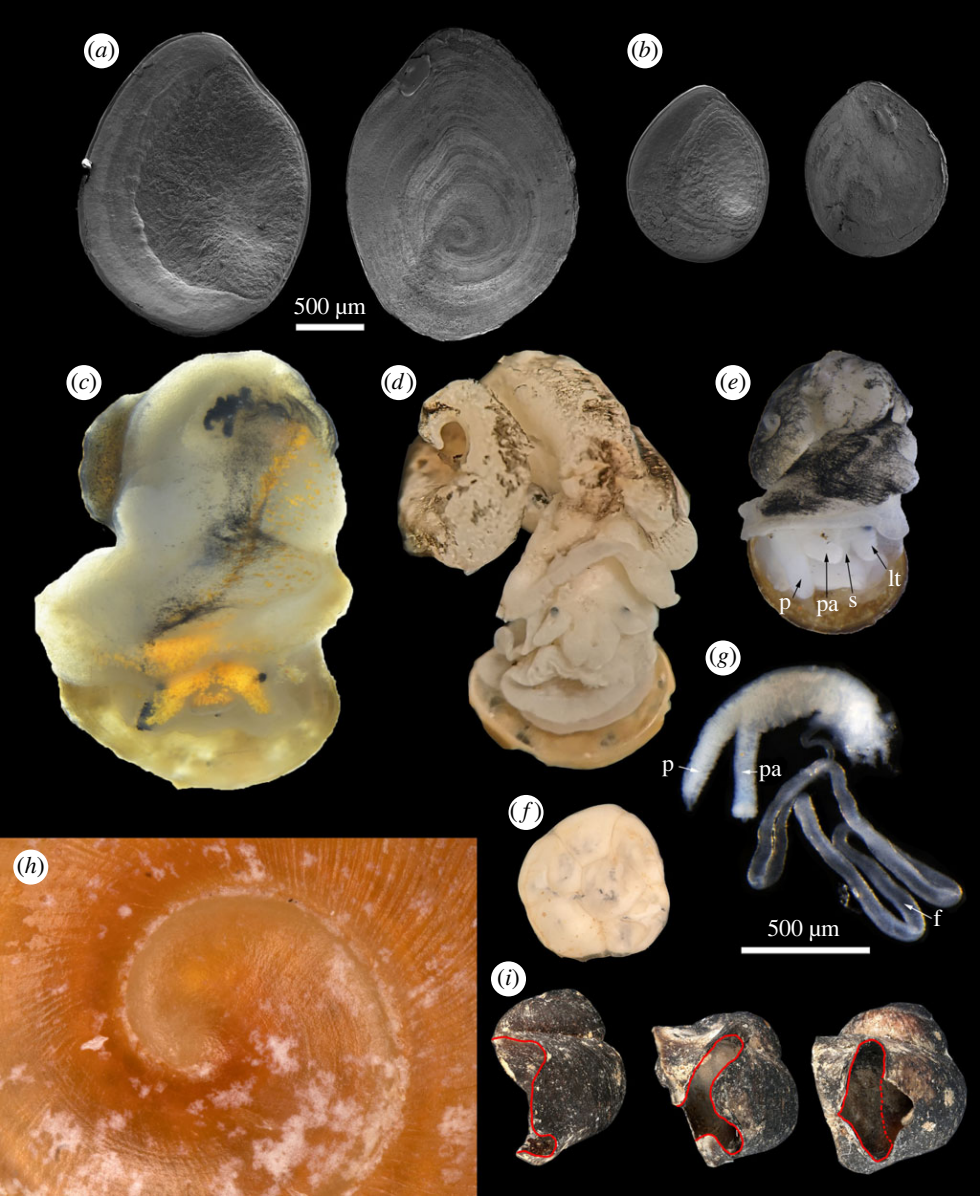
Morphological details of *Helicostoa liuae* sp. nov. (*a*) SEM photo of the female operculum, left is interior surface and right is exterior surface. (*b*) SEM photo of the male operculum, left is interior surface and right is exterior surface. (*c*) Fresh soft body of female. (*d*) Ethanol preserved soft body of female. (*e*) Ethanol preserved soft body of male. (*f*) Egg capsules. (*g*) Male genital. (*h*) Protoconch of female, photographed by Bernhard Schurian. (*i*) Different stages of sub-adult females showing the enclosing process of the opening. (*a–f*) Scale bar 500 µm; (*g*) scale bar 500 µm. Abbreviations: p, penis; pa, penial appendix; s, snout; lt, left tentacle; f, flagellum.

Protoconch (figure 5*h*) broad, low dome-shaped, nucleus of protoconch relatively smooth, the rest of protoconch with fine granule and several spiral striations; no other obvious sexual dimorphism except for the size.

Operculum (figure 5*a*–*b*) calcareous, ovate, thin and fragile, transparent white in colour; on exterior surface a paucispiral nucleus visible, with one anti-clockwise whorl, located on the lower central part, concentric growth lines surrounding the nucleus; on interior surface a large inner opercular region visible, with many grains and veins; no other obvious sexual dimorphism except for the size.

Head, foot and mantle of living animals (figure 5*c*) transparent white or grey, with fine dense yellow pigment spots on the upper side of snout, tentacles, region around eyes and mantle edge, and sparse yellow pigment spots and dense fine black spots on the mantle; ethanol-preserved specimens (figure 5*d*–*e*) with pale yellow body colour and irregular black spots on the surface of mantle.

Male genital (figure 5*g*) with very long flagellum, about five times as the length of penis; the thickness of penis uniform, tip pointed, length of penial appendix same as that of distal part (from the base of penial appendix to the tip of penis) of penis, length of distal part of penis same as that of proximal part (from the base of penial appendix to the base of penis) of the penis; penis situated in the neck behind the right tentacle of male.

Egg capsules kept in ethanol (figure 5*f*) white, with a group size of 6 individuals, clustering together tightly into a round flat shape.

Radula (*n* = 5, 3 females and 2 males; electronic supplementary material, figure S3) oblong. Central teeth with 5 to 6 cusps in females or 4 cusps in males on either side of median cusp, median cusp with wider base longer than adjacent cusps; 3 to 4 cusps on either lateral edge of central teeth, inner pair much larger than others. Lateral teeth with 5 to 7 cusps in females or 5 to 6 cusps in males on either side of median cusp, median cusp with wider base longer than adjacent cusps; inner lateral edge of lateral teeth with a concave on lower part, only half of outer lateral edge in length. Marginal teeth slender, slightly oblong; inner marginal teeth with 25 to 41 cusps in females or 29 to 43 cusps in males, the fourth or fifth cusp in females or sixth to seventh cusp in males counting from outer side larger than the other cusps; outer marginal teeth with 23 to 24 cusps in females or 23 to 27 cusps in males.

**Morphological comparisons:** the shell of *Helicostoa liuae* sp. nov. displays a striking sexual dimorphism, which is related to different life histories of males and females. The shell of the sessile adult female can be distinguished from that of the vagile adult male based on its much larger size, more whorls, higher spiral, closed primary aperture, and with a bow-shaped opening. The sessile adult female can be distinguished from *Helicostoa sinensis* based on its much smaller size, conical shell, a wide bow-shaped opening and ovate operculum. The shell of vagile adult male is most similar to that of another bithyniid species *Sierraia expansilabrum* Brown, 1988 from Sierra Leone, which has a small globose shell and extended labral margin. However, it can be easily distinguished from species of *Sierraia* based on its much smaller oblate orange shell, operculum characters and many more cusps on the marginal teeth of radula. The shell of juvenile female of *Helicostoa liuae* sp. nov. can be distinguished from that of the juvenile male based on its higher spire.

**Ecology:** this species is mainly found on the surface of limestone rocks in big rivers, but it sometime also can be found attached on bricks and snail or mussel shells in big rivers; adult females are sessile and adult males are vagile (figure 1*f*); sessile adult females produce egg capsules in the cavity of shell, and capsules cluster together, tightly attaching to the sealed aperture. When kept in aquarium, the sessile females could actively do suspension feeding; the red form of living planktonic algae *Chlamydomonas reinhardtii* was accepted as food by the snails, and the snails excrete red faeces (figure 1*g*); the sessile females could stretch out from the secondary aperture (figure 1*e*), but normally only stay close to the secondary aperture in the shell (figure 1*g*).

**Distribution:** the species currently is only found in Long River (龙江), Qian River (黔江) and Xun River (浔江) in Guangxi Zhuang Autonomous Region, China. However, since this species was found syntopic with *Helicostoa sinensis*, it apparently shares or used to share at least parts of its distribution range with *Helicostoa sinensis*.

## 4. Discussion

### (a) Taxonomy and evolutionary innovation

In this study, we rediscovered the ‘T type’ of enigmatic sessile freshwater snail *Helicostoa* lost for a century. In agreement with the results of Wilke *et al*. [16], our phylogeny based on COI and 16S supports the assignment of genus *Helicostoa* to the family Bithyniidae. Therefore, the family Helicostoidae is a junior synonym of Bithyniidae. In addition, morphological and anatomical characters also strongly support that *Helicostoa* is a memeber of Bithyniidae. The structure of the male genital (figure 5*g*) is quite similar to that of *Bithynia* [26]; the radula (electronic supplementary material, figure S3) displays a typical structure found in Bithyniidae; the calcified operculum (figure 3*i*; figure 5*a*–*b*) is also typical for Bithyniidae [27–29]. The subfamily-level taxonomy of *Helicostoa* within the new subfamily Helicostoinae proposed by Wilke *et al*. [16] should be revised based on a more integrative study of Bithyniidae involving more genera and species in the future.

The molecular phylogeny strongly suggests that the ‘T type’ should be a different species from the ‘P type’. The ‘P type’ now is designated as *H. sinensis*, and the ‘T type’ is described as a new species *H. liuae*. The p-distance of COI and 16S between *H. sinensis* and *H. liuae* support that they should be two different species within one genus. No genetic differences were found between two sequenced populations of *H. liuae* that are geographically far apart. As for morphological characters, *H. sinensis* can be easily distinguished from both genders of *H. liuae* based on its discoidal shape, much larger size, round opening and round operculum, which is consistent with the results of Lamy [8] and Pruvot-Fol [9]. We did not find a jaw in specimens of *H. liuae*, which was mentioned by Pruvot-Fol [9] for the ‘T type’. Since a jaw has never been observed in Bithyniidae, we suspect that Pruvot-Fol misidentified this character.

The sessile individuals of *Helicostoa* species cannot be confused with any other species of superfamily Truncatelloidea, especially when considering the opening on the body whorl. The opening on the body whorl of sessile *H. liuae* is wide bow-shaped, and that of *H. sinensis* is round. The growing process of this opening can be seen in several juvenile shells (figure 5*i*). Based on aquarium observations, the opening of *H. liuae* take over the function of the aperture sealed by shell matter and stone. Sessile *H. liuae* could stretch their heads or tentacles out, secret mucus, do ingestion and excretion from this secondary aperture (figure 1*e*,*g*). Although we have not observed the living *H. sinensis*, we suspect that the opening of sessile *H. sinensis* should play the same role as that of *H. liuae*.

The morphology of this opening in *Helicostoa* is somehow similar to that of selenizone in a few marine snail species of Scissurelloidea Gray, 1847, such as *Satondella danieli* Segers, Swinnen & Abreu, 2009. The selenizone of Scissurelloidea is a slit or a single opening on the body whorl, with the main function of respiration [30]. However, this similarity is obviously the result of convergent evolution, since there is no close relationship between Bithyniidae of Caenogastropoda and Scissurelloidea of Vetigastropoda. The single opening on the body whorl working as the aperture seems like a unique evolutionary innovation of *Helicostoa* within entire Mollusca.

### (b) Sessility and sexual dimorphism

Sessility is an extremely rare character within freshwater gastropods. Except for species of *Helicostoa*, *Melanoides agglutinans* is the only other known freshwater gastropod species with a sessile habit. *M. agglutinans* was only found in crevices of rocks immersed in swift water from lower Congo River [31]. *M. agglutinans* seems to adhere foreign objects at any part of its shell where the growing is impeded, and becomes deformed at the sessile part. Bequaert & Clench [31] considered that the deformation and sessility of this species would be probably lost if the young snails were moved from crevices to a less restricted environment, and ‘normal’ adult shells might be obtained. Besides, there is no sexual dimorphism observed in shell morphology and sessility within this species.

Species of *Helicostoa* display a completely different strategy of sessility from *Melanoides agglutinans*, as sessile females of both *H. liuae* and *H. sinensis* have a specific attachment area instead of attaching randomly to the substrate as *Melanoides agglutinans*. However, the specific attachment areas of the two *Helicostoa* species are different. *H. liuae* attaches itself only at the sealed aperture of the last whorl (figure 4*a*), while *H. sinensis* fixes itself on stones tightly from the base of the second whorls (figure 3*g*) to the sealed aperture. As for *H. sinensis,* the area attaching to the substrate can be quite flat (figure 3*h*), and the body whorl can become open-coiled when it meets an obstacle (figure 3*f*)*.* This difference of attachment area suggests that *H. sinensis* should have an earlier and longer sessile period in life history compared to *H. liuae*. The SEM images of the sessile area of *H. liuae* show that the shell incorporates foreign objects into shell matter, such as micro-tube (electronic supplementary material, figure S1*b*–*g*) or irregular particles (electronic supplementary material, figure S1j–l). The micro-tubes with their soft, smooth wall and uniform diameter are probably not a structure of the shell but some microorganism like Cyanobacteria.

Sessility in females of *Helicostoa* might arguably be linked to reproduction and have evolved as a form of maternal care, as the females are also brooding (i.e. retaining the eggs within their shells). It might be speculated that this prevents eggs from being swept away by the swift water flow in the habitat of *Helicostoa*. However, egg capsules of other gastropods (*Hua* spp. of family Semisulcospiridae) have been observed on rocks in the same habitats. Moreover, bithyniid species of *Sierraia* endemic to Sierra Leone dwell in a similar habitat on the rock surface in rapidly flowing rivers, but they can move freely and lay their eggs directly onto the surface of other shells [27]. Therefore, there is no clear evidence to support this hypothesis at present. More detailed ecological studies of living animals are necessary to understand its adaption of sessility.

In addition, we discovered a remarkable sexual dimorphism in *H. liuae*. We surprisingly found that all sessile individuals of *H. liuae* are females. Some free-moving micro snails from the same locality were shown to be the males of *H. liuae* based on DNA sequences (figure 2) and characters of the radula (electronic supplementary material, figure S3). The differences of morphology and habits between males and females of *H. liuae* are so significant that we initially considered them to be different species. *H. liuae* displays its remarkable sexual dimorphism in shell size, opening structure on the body whorl, and habit of sessility. The female of *H. liuae* is about two to three times larger than the male (table 1 W, H), and the male is much flatter than the female (table 1 W/H).

The larger size of females can be explained by fecundity selection, especially given that eggs are retained in the shell. However, the driving mechanism of sexual dimorphism in opening structure and habit of sessility is more complicated. The opening structure of sessile females, which has been discussed above, is probably an evolutionary innovation triggered by sessility, since the original aperture is sealed to fix the shell to the substrate. The vagile males can easily search for and copulate with different sessile females. Pruvot-Fol also considered that vagile males would be advantageous for mating [9], where sessility is an obvious disadvantage. The marine sessile gastropod *Vermicularia spirata* (Philippi, 1836) is a protandrous hermaphrodite [32]. The free-living small males can search for and mate with sessile large females, which were males before completing sex reversal. Within Truncatelloidea, protandrous hermaphroditism is only reported from one marine species of the family Vitrinellidae [33]. We consider that *Helicostoa liuae* has evolved an extreme sexual dimorphism instead of protandrous hermaphroditism to overcome the obstacles in mating imposed by sessility. Both the secondary aperture and sexual dimorphism seem to be adaptions to its sessile life history. More studies, including the systematic revision of family Bithyniidae to find the sister group of *Helicostoa*, and ecological study of *Helicostoa*, can help us to better understand the evolution of such a remarkable sexual dimorphism.

As for *H. sinensis*, we strongly suspect that it should have a similarly striking sexual dimorphism. All the sessile individuals of *H. sinensis* are probably females as those of *H. liuae*. However, this assumption can only be tested by finding living *H. sinensis*.

### (c) Habitat and distribution

With the discovery of the new *Helicostoa* species, three species of sessile freshwater gastropods are known to science. All of them are attached to rocks in large rivers: Congo River for *M. agglutinans*, Yangtze River (doubtful, see following) for *H. sinensis*, and Pearl River for *H. liuae*.

*H. liuae* is discovered in three large tributaries of the Pearl River Basin: Long, Qian and Xun River. Qian and Xun River are the main arms of the Pearl River. The average flow of Xun River is 7271 m^3^ s^−1^, and the largest flow of it can reach 44 900 m^3^ s^−1^ [34]. The type locality of *H. liuae* in Long River has a much lower average flow, about 387 m^3^ s^−1^, but its largest flow can also reach 10 400 m^3^ s^−1^ [35]. Long River is the upstream river of Liu River, and Liu River is the upstream river of Qian River. The distance between the two collecting localities in Long and Qian along the river course is more than 300 km. In contrast to *M. agglutinans* inhabiting crevices, the two *Helicostoa* species both dwell on the rock surface. These limestone rocks in big rivers seem to offer a special niche for *Helicostoa*. Although we found some other species of Pomatiopsidae on the rocks, this sessile *Helicostoa* species is undoubtedly the dominant species and shows a special adaptation to this habitat.

Since we did not discover any living or dead *H. sinensis*, the distribution of *H. sinensis* remains enigmatic. However, the coexisting of *H. sinensis* and *H. liuae* on the same type-bearing rocks kept in Paris has already demonstrated that the distributions of two species must overlap to some degree. The distribution of *H. liuae*, the Pearl River Basin, is far from the main arm of Yangtze River. The two river basins are only artificially connected by a canal called Lingqu built during the Qin Dynasty of China (219 BCE). The type locality information of *H. sinensis*, ‘Kouei-Tchéou’ in French, was based on an around 20 years' old note, not directly recorded by Lamy [8]. It could refer to ‘夔州’ (Kuizhou, current Fengjie County near Three Gorges region) or ‘贵州’ (Guizhou Province).

The Three Gorges region fits the description of ‘more than 1200 km away from Shanghai’. However, neither living animals or dead shells of *H. sinensis* have ever been recorded from Yangtze River in the Three Gorges region since the description, even though there have been several comprehensive surveys of freshwater molluscs conducted by experts before the construction of the Three Gorges Dam [14,15]. Since *H. sinensis* seems like an abundant or even dominant species fixed to the rock based on the types (figure 3*a,b*), it is strange that not even one empty shell has ever been discovered, if this species was really distributed there. In comparison, we discovered large amounts of empty shells of *H. liuae* on the bank of river at the type locality.

Meanwhile, the Long River, where the type locality of *H. liuae* is located, originates from Guizhou Province (figure 1*a*). It is possible to find *H. liuae* in Guizhou as well. Besides, the Yangtze River Basin and the Pearl River Basin are adjacent in Guizhou Province. Considering the sympatry of two *Helicostoa* species, we strongly suspect that the rocks with the types of *H. sinensis* were collected from a river in Guizhou Province. To raise the possibility finding *H. sinensis*, we suggest that more surveys should be conducted on the rocks from rivers in Guizhou Province in the future.

Freshwater fauna is easily impacted by human activities, such as overharvesting, pollution, transportation, sand mining, damming and riverbank construction. Freshwater gastropods represent some of the most threatened species, with nearly 20% of recorded extinctions within molluscs according to IUCN [36]. Many freshwater gastropods from SW China are facing dramatic population declines [37,38]. *H. liuae* is a relatively common species at the collecting sites in the Long, Qian and Xun River. However, all of these sites are very close to urban area. Many dams have been constructed near the habitats of *H. liuae*, such as the Datengxia Hydropower Station in the Qian River [39]. These factors add high environmental risks to the habitat of this sessile species. Considering the two populations without genetic difference are more than 300 km far apart, *H. liuae* should have a relatively strong dispersal capability in the rivers. More surveys should be conducted in the connecting Liu, Hongshui and Yu River to investigate whether more populations of *H. liuae* exist and to what degree they are continuous.

## Data Availability

The datasets supporting this article have been uploaded as part of the electronic supplementary material [40].

## References

[1] Darwin C. 1888 The descent of man, and selection in relation to sex. London, UK: John Murray.

[2] Páll-Gergely B, Hunyadi A, Otani JU, Ablett JD, Schilthuizen M. 2020 First record of striking sexual dimorphism in two terrestrial caenogastropods. J. Mollusc. Stud. **86**, 254-258. (10.1093/mollus/eyaa005)

[3] Uvayeva O, Vakaliuk T, Shcherbina G, Shimkovich E. 2021 Sexual dimorphism in shell morphology of mollusks of the genus Viviparus: important objects of water resources of Ukraine. In *E3S Web of Conferences 280*: EDP Sciences.

[4] Uba K. 2019 Sexual dimorphism, asymmetry, and allometry in the shell shape of *Modiolus metcalfei* (Hanley, 1843) collected from Dumangas, Iloilo, Philippines: a geometric morphometric approach. Computat. Ecol. Softw. **9**, 107.

[5] Pastorino G. 2007 Sexual dimorphism in shells of the southwestern Atlantic gastropod *Olivella plata* (Ihering, 1908) (Gastropoda: Olividae). J. Mollusc. Stud. **73**, 283-285. (10.1093/mollus/eym024)

[6] Clark SA, Miller AC, Ponder WF. 2003 Revision of the snail genus *Austropyrgus* (Gastropoda: Hydrobiidae): a morphostatic radiation of freshwater gastropods in southeastern Australia. Rec. Austral. Museum **28**, 1-109. (10.3853/j.0812-7387.28.2003.1377)

[7] Pascual MS, Iribarne OO, Zampatti EA, Bocca AH. 1989 Female-male interaction in the breeding system of the puelche oyster *Ostrea puelchana* d'Orbigny. J. Exp. Mar. Biol. Ecol. **132**, 209-219. (10.1016/0022-0981(89)90128-7)

[8] Lamy E. 1926 Sur une coquille enigmatique. Journal de Conchyliologie **70**, 51-56.

[9] Pruvot-Fol A. 1937 Étude d'un prosobranche d'eau douce: *Helicostoa sinensis*. Bulletin de la Société zoologique de France **62**, 250-257.

[10] Bequaert J, Clench WJ. 1941 Concerning gastropods adhering to foreign objects. Science **94**, 514. (10.1126/science.94.2448.514.a) 17809181

[11] Heppell D. 1995 Helicostoa: a forgotten Chinese gastropod enigma. In Abstracts, Twelfth International Malacological Congress, pp. 29-30. Vigo, Spain.

[12] Bouchet P, Rocroi JP, Hausdorf B, Kaim A, Kano Y, Nützel A, Parkhaev P, Schrödl M, Strong EE. 2017 Revised classification, nomenclator and typification of gastropod and monoplacophoran families. Malacologia **61**, 1-526. (10.4002/040.061.0201)

[13] Wilke T. 2019 17. *Helicostoidae* Pruvot-Fol, 1937. In Freshwater mollusks of the world (eds C Lydeard, KS Cummings), pp. 109-110. Baltimore, MD: Johns Hopkins University Press.

[14] Liu YY, Wang YX, Zhang WZ. 1991 On the freshwater molluscs in the area of Sanxia Reservoir. Acta Zootaxonomica Sinica **16**, 1-14.

[15] Davis GM, Wilke T, Spolsky C, Qiu CP, Qiu DC, Xia MY, Zhang Y, Rosenberg G. 1998 Cytochrome oxidase I-based phylogenetic relationships among the Pomatiopsidae, Hydrobiidae, Rissoidae and Truncatellidae (Gastropoda: Caenogastropoda: Rissoacea). Malacologia **40**, 251-266.

[16] Wilke T, Kehlmaier C, Stelbrink B, Albrecht C, Bouchet P. 2023 Historical DNA solves century-old mystery on sessility in freshwater gastropods. Mol. Phylogenet. Evol. **185**, 107813. (10.1016/j.ympev.2023.107813) 37187366

[17] Zhang LJ, von Rintelen T. 2021 The neglected operculum: a revision of the opercular characters in river snails (Caenogastropoda: Viviparidae). Journal of Molluscan Studies **87**, eyab008. (10.1093/mollus/eyab008)

[18] Vrijenhoek R. 1994 DNA primers for amplification of mitochondrial cytochrome c oxidase subunit I from diverse metazoan invertebrates. Mol. Mar. Biol. Biotech. **3**, 294-299. 7881515

[19] Schultheiß R, Wilke T, Jørgensen A, Albrecht C. 2011 The birth of an endemic species flock: demographic history of the *Bellamya* group (Gastropoda, Viviparidae) in Lake Malawi. Biol. J. Linnean Soc. **102**, 130-143. (10.1111/j.1095-8312.2010.01574.x)

[20] Palumbi SR, Martin A, Romano S, McMillan WO, Stice L, Grabowski G. 1991 The simple fools guide to PCR. Honolulu, HI: University of Hawaii Press.

[21] Bunchom N et al. 2021 Genetic structure and evidence for coexistence of three taxa of *Bithynia* (Gastropoda: Bithyniidae), the intermediate host of Opisthorchis viverrini sensu lato (Digenea: Opisthorchiidae) in Thailand examined by mitochondrial DNA sequences analyses. Acta Trop. **221**, 105980. (10.1016/j.actatropica.2021.105980) 34048791

[22] Edgar RC. 2004 MUSCLE: multiple sequence alignment with high accuracy and high throughput. Nucleic Acids Res. **32**, 1792-1797. (10.1093/nar/gkh340) 15034147 PMC390337

[23] Kumar S, Stecher G, Li M, Knyaz C, Tamura K. 2018 MEGA X: molecular evolutionary genetics analysis across computing platforms. Mol. Biol. Evol. **35**, 1547. (10.1093/molbev/msy096) 29722887 PMC5967553

[24] Stamatakis A. 2014 RAxML version 8: a tool for phylogenetic analysis and post-analysis of large phylogenies. Bioinformatics **30**, 1312-1313. (10.1093/bioinformatics/btu033) 24451623 PMC3998144

[25] Ronquist F et al. 2012 MrBayes 3.2: efficient Bayesian phylogenetic inference and model choice across a large model space. Syst. Biol. **61**, 539-542. (10.1093/sysbio/sys029) 22357727 PMC3329765

[26] Kim JJ. 2005 Comparative anatomy of the family Bithyniidae (Prosobranchia: Mesogastropoda). The Korean Journal of Malacology **21**, 133-145.

[27] Brown DS. 1988 *Sierraia*: rheophilous West African river snails (Prosobranchia: Bithyniidae). Zoological Journal of the Linnean Society **93**, 313-355. (10.1111/j.1096-3642.1988.tb01366.x)

[28] Ponder WF. 2003 Monograph of the Australian Bithyniidae (Caenogastropoda: Rissooidea). Zootaxa **230**, 1-126. (10.11646/zootaxa.230.1.1)

[29] Glöer P, Pešić V. 2006 On the identity of *Bithynia graeca* Westerlund, 1879 with the description of three new *Pseudobithynia* n. gen. species from Iran and Greece (Gastropoda: Bithyniidae). Malakologische Abhandlungen **24**, 29-36.

[30] Geiger DL. 2012 Monograph of the little slit shells. Santa Barbara, CA: Santa Barbara Museum of Natural History.

[31] Bequaert JC, Clench WJ. 1941 Additions to the rheophilous mollusk fauna of the Congo estuary. Cambridge, MA: Museum of Comparative Zoology.

[32] Bieler R, Hadfield MG. 1990 Reproductive biology of the sessile gastropod *Vermicularia spirata* (Cerithioidea: Turritellidae). J. Molluscan Studies **56**, 205-219. (10.1093/mollus/56.2.205)

[33] Bieler R, Mikkelsen PM. 1988 Anatomy and reproductive biology of two western Atlantic species of Vitrinellidae, with a case of protandrous hermaphroditism in the Rissoacea. Nautilus **102**, 1-29.

[34] Annals of Guiping County compilation committee. 1991 Annals of guiping county. Nanning, China: Guangxi People's Publishing House.

[35] Annals of Yizhou City compilation committee. 1998 Annals of yizhou city. Nanning, China: Guangxi People's Publishing House.

[36] Strong EE, Gargominy O, Ponder WF, Bouchet P. 2007 Global diversity of gastropods (Gastropoda; Mollusca) in freshwater. In Freshwater animal diversity assessment, pp. 149-166. Dordrecht, The Netherlands: Springer.

[37] Zhang LJ, Chen SC, Yang LT, Jin L, Köhler F. 2015 Systematic revision of the freshwater snail *Margarya* Nevill, 1877 (Mollusca: Viviparidae) endemic to the ancient lakes of Yunnan, China, with description of new taxa. Zoological Journal of the Linnean Society **174**, 760-800. (10.1111/zoj.12260)

[38] Zhang LJ. 2017 A new species of freshwater snail *Tchangmargarya* (Gastropoda: Viviparidae) endemic to a vanished small lake in Yunnan, China. Molluscan Research **37**, 252-257. (10.1080/13235818.2017.1323369)

[39] Lu K. 2020 Study on Hydrologic Forecasting at the Construction Period of Datengxia Hydroproject. Proceedings of the International Association of Hydrological Sciences **383**, 93-97. (10.5194/piahs-383-93-2020)

[40] Zhang L-J, Shi Z-A, Chen Z-Y, von Rintelen T, Zhang W, Lou Z-J. 2024 Rediscovery and systematics of the enigmatic genus *Helicostoa* reveals a new species of sessile freshwater snail with remarkable sexual dimorphism. Figshare. (10.6084/m9.figshare.c.6978832) PMC10777140 38196368

